# Paired CycleGAN-based virtual staining for 3D X-ray histology of bone-implant systems

**DOI:** 10.1107/S1600577526004340

**Published:** 2026-06-05

**Authors:** Sarah C. Irvine, Christian Lucas, Diana Krüger, Bianca C. Guedert, Julian Moosmann, Berit Zeller-Plumhoff

**Affiliations:** a Institute of Materials Physics Helmholtz-Zentrum Hereon 21502Geesthacht Germany; b Institute of Metallic Biomaterials Helmholtz-Zentrum Hereon 21502Geesthacht Germany; c Bruker Daltonics SPR, 20251Hamburg, Germany; d Data-Driven Analysis and Design of Materials University of Rostock 18059Rostock Germany; Tohoku University, Japan

**Keywords:** virtual staining, 3D X-ray histology, virtual histology, biodegradable bone implants, paired CycleGAN

## Abstract

Three-dimensional X-ray histology offers a non-invasive alternative to conventional 2D histology, enabling volumetric imaging of biological tissues without physical sectioning or chemical staining. However, the intrinsic greyscale contrast of X-ray tomography limits its biochemical specificity compared with traditional histological stains. In this study, we extend deep-learning-based virtual staining to the X-ray domain via cross-modality image translation to generate artificially stained slices directly from synchrotron radiation microtomography (µCT) scans. Using over 50 co-registered pairs of µCT and toluidine blue-stained histology from bone-implant samples, we trained a modified CycleGAN network tailored for limited paired data. Whole-slide histology images were downsampled to the CT voxel size, with on-the-fly data augmentation for patch-based training. The model incorporates pixelwise supervision and greyscale consistency losses, enabling histologically realistic colour outputs while preserving structural detail. Results outperformed Pix2Pix and standard CycleGAN baselines across metrics of structural similarity, perceptual fidelity, and peak signal-to-noise ratio. Once trained, the model can be applied to full µCT volumes to produce virtually stained 3D datasets that enhance interpretability without additional sample preparation. This work introduces virtual staining to 3D X-ray imaging, which may provide a scalable route for chemically informative, label-free tissue characterization in biomedical research.

## Abstract 2

This work demonstrates virtual staining for 3D X-ray histology using a modified CycleGAN trained on paired synchrotron radiation microtomography (SR-µCT) and histology images of bone-implant specimens. The method enables the generation of artificially coloured contrast resembling conventional histological staining from label-free SR-µCT data and is scalable across entire 3D volumes without additional sample preparation.

## 1. Introduction

Histological analysis is widely regarded within clinical pathology and biomedical research as the benchmark technique for characterizing tissue architecture and cellular detail. Through chemical staining of thin sections and brightfield microscopy, conventional histology provides high specificity in identifying cell types, pathological states, and tissue organization. However, this process is inherently destructive, labour-intensive, and fundamentally two-dimensional (Abraham & Levenson, 2024 ▸). While serial sectioning can be used to reconstruct three-dimensional structures, it remains time-consuming, prone to alignment artefacts, and yields anisotropic spatial resolution due to finite section thickness and non-contiguous through-plane sampling (Pichat *et al.*, 2018 ▸). As a result, conventional histology captures only limited volumetric context, posing significant challenges for studying complex 3D tissue and biomaterial interactions, particularly in whole organs or biomedical implants.

Overcoming those limitations, three-dimensional (3D) X-ray imaging techniques such as synchrotron radiation X-ray micro-tomography (SR-µCT) and high-resolution laboratory µCT present as powerful alternatives. These methods enable label-free, non-invasive visualization of soft and mineralized tissues at micrometre-scale resolution. When applied correlatively with histology for reference, such approaches may be collectively termed ‘3D X-ray histology’ or ‘X-ray virtual histology’ (Albers *et al.*, 2018 ▸; Töpperwien *et al.*, 2018 ▸; Katsamenis *et al.*, 2019 ▸). By providing volumetric datasets that retain cellular- and tissue-level detail across intact specimens, these techniques have the potential to support a transition toward 3D pathology, enabling more comprehensive characterization of morphology across entire tissue volumes (Song *et al.*, 2024 ▸). However, despite these advantages, a central limitation of 3D X-ray histology is the lack of biochemical specificity: image contrast is intrinsically greyscale, derived from differences in X-ray attenuation or phase shift, and does not replicate the cell-type or structure-specific colour-coded information provided by traditional histological stains. While X-ray contrast agents based on high-*Z* elements can be applied to enhance soft tissue visibility, these typically bind to tissue components differently than conventional stains, and the issue of bio-specificity remains unresolved. Some progress has been made through the development of modified histological stains that incorporate heavy metal complexes, offering improved compatibility with traditional staining protocols (Müller *et al.*, 2018 ▸; Petzold *et al.*, 2024 ▸). However, the heavy-metal related issues of toxicity, environmental safety, and clinical applicability are of some concern. As a result, computational methods that enhance the interpretability of raw X-ray volumes without the need for physical staining represent a valuable and increasingly important advancement. In one early X-ray histology study (Khimchenko *et al.*, 2016 ▸), a joint histogram-based analysis was used to map greyscale CT intensities to histological colour values, enabling stain-like colourization of 3D volumes. However, this approach has seen limited uptake in subsequent research.

Recent advances in deep learning, in particular the development of generative adversarial networks (GANs), have transformed the field of computational image synthesis, including applications of digital pathology. GANs are composed of two competing neural networks: a generator that synthesizes images and a discriminator that evaluates their realism, trained adversarially to produce outputs indistinguishable from ground-truth data (Goodfellow *et al.*, 2014 ▸). In medical imaging, GANs have been applied to tasks such as segmentation, super-resolution, denoising, artefact reduction, and, notably, cross-modality image-to-image translation, where a transformation is learned between different imaging modalities (Yi *et al.*, 2019 ▸). Examples include synthesizing computed tomography (CT) from magnetic resonance imaging (MRI) or positron emission tomography (PET) from CT, which are clinically valuable when certain modalities are limited by cost, radiation exposure or accessibility.

‘Virtual staining’ refers to a family of deep-learning approaches that are becoming increasingly established in computational histopathology research, whereby label-free microscopy images are transformed into the visual equivalent of chemically stained histological images, for enhanced interpretability without the need for physical staining (Latonen *et al.*, 2024 ▸). These primarily GAN-based techniques have demonstrated high-fidelity image-to-image translation, applied within various optical imaging modalities including autofluorescence (Rivenson *et al.*, 2019 ▸), photo-acoustic microscopy (Kang *et al.*, 2022 ▸) and quantitative phase imaging (QPI) (Abraham *et al.*, 2022 ▸), as well as standard brightfield microscopy (Koivukoski *et al.*, 2023 ▸). While some of these optical approaches permit limited volumetric acquisition without physical sectioning, they typically require either thin (sub-mm) specimens or optically transparent samples via tissue clearing. To date, virtual staining has not yet been extended to the X-ray domain, where the image formation mechanisms and contrast properties differ significantly. In this study, we introduce a deep-learning model for virtual staining in 3D X-ray histology, enabling direct synthesis of virtually stained 3D whole tissue volumes from raw greyscale µCT scans. This cross-modality translation aims to combine the structural integrity of X-ray imaging with histologically meaningful contrast.

For the base model architecture, we consider as reference two foundational models in image-to-image translation: Pix2Pix (Isola *et al.*, 2017 ▸) and CycleGAN (Zhu *et al.*, 2017 ▸), representing supervised and unsupervised approaches, respectively. Supervised models like Pix2Pix require spatially aligned paired datasets, which are often difficult or even impossible to obtain in medical imaging due to motion, resolution mismatch or other acquisition constraints. CycleGAN addresses this limitation through a cycle-consistency loss that enables training with unpaired data. In earlier applications of GANs in medical imaging, Pix2Pix, CycleGAN and variants thereof comprised a majority of cross-modality synthesis tasks (Yi *et al.*, 2019 ▸), although as the field has progressed there has been a shift towards increasingly task-specific GAN models tailored to address the unique challenges of modality translation and clinical relevance (Heng *et al.*, 2024 ▸). CycleGAN remains the most widely adopted unsupervised approach in virtual staining, where fully aligned datasets are rarely available (Latonen *et al.*, 2024 ▸). In the related context of virtual stain transfer, where one histochemical stain is virtually transformed into another, CycleGAN was found to outperform other architectures, including Pix2Pix, even in settings where simulated paired data were available (Zingman *et al.*, 2024 ▸).

Pix2Pix may still yield superior results when perfect alignment between modalities is available, whereas the un­supervised CycleGAN is ideal for situations with no assumed alignment. Many real-world medical imaging scenarios, however, fall between these extremes, with some degree of partial misalignment. This intermediate setting has motivated adaptations of CycleGAN for use with paired data, retaining its robust architecture while benefiting from supervision. The added stability offered by cycle-consistency constraints encourages learning of mappings that are reversible and coherent across imperfectly aligned image domains (Kaji & Kida, 2019 ▸). Several studies have explored such paired CycleGAN frameworks, including work on cone-beam CT correction and dual-energy chest X-ray imaging, where mis­aligned data are actively corrected (Harms *et al.*, 2019 ▸; Ueda *et al.*, 2025 ▸), as well as MRI-to-CT synthesis (Lei *et al.*, 2019 ▸) and contrast-enhanced mammography (Rofena *et al.*, 2024 ▸). These studies illustrate the flexibility of CycleGAN variants in modality translation tasks where precise pixel-wise alignment is lacking but a high level of spatial correspondence is preserved. Our work adopts a similar approach, employing a paired CycleGAN framework adapted to the X-ray and histology domains, and incorporating additional supervisory loss terms to improve structural fidelity in domain mapping. We evaluate the performance of this modified CycleGAN in comparison with both the standard CycleGAN and Pix2Pix models.

**Figure 1:**
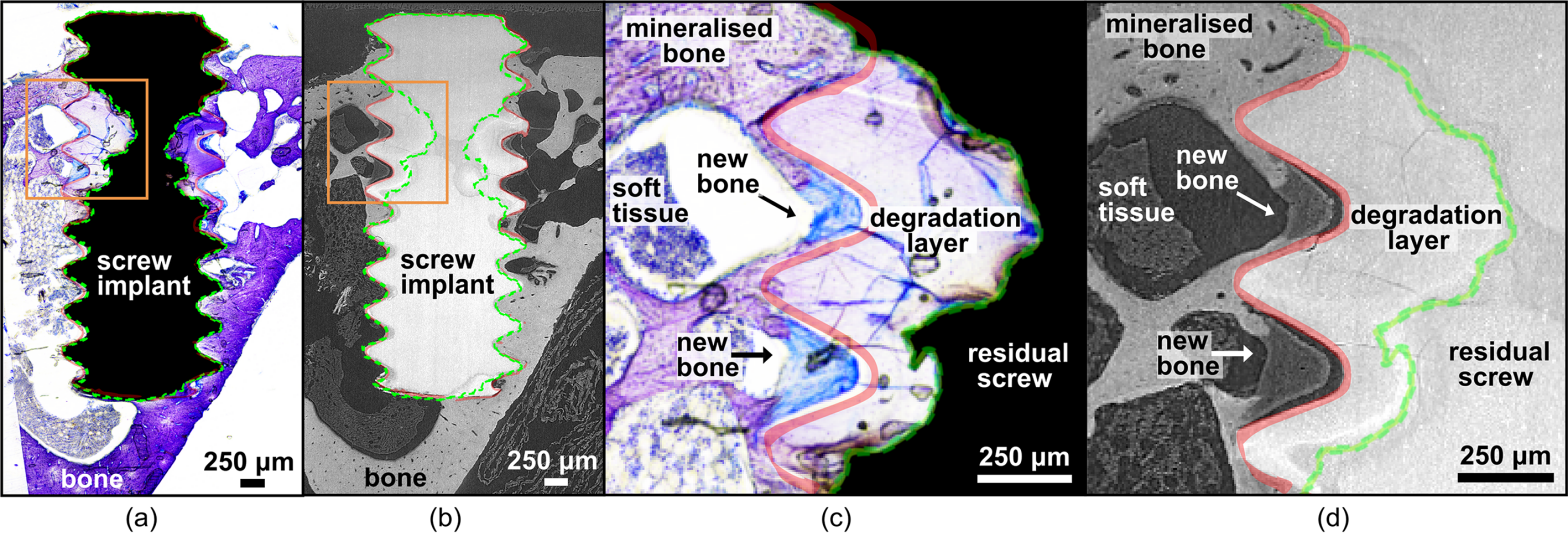
Illustrative example of our X-ray-histology application, with (real) paired toluidine-blue stained histology (*a*) and SR-µCT (*b*) images of an Mg–10Gd screw implant in bone extracted after eight weeks *in vivo*. In the histology, the green line marks the interface between the residual screw and degradation layer. In the µCT slice, the red line indicates the boundary between the degradation layer and surrounding bone. The residual screw appears black in histology due to total light attenuation by the metal. Regions identified as the degradation layer in histology correspond to areas in µCT with mildly differing X-ray attenuation to the residual screw. Higher magnification views (*c*, *d*) indicated by the orange boxes highlight the areas surrounding the degradation layer. Arrows in black (*c*) or white (*d*) point to regions of new (woven) bone, appearing blue in histology and exhibiting lower attenuation values in µCT due to lower mineralization content. Figure adapted from Krüger *et al.* (2022) ▸ under a CC-BY-4.0 licence.

To demonstrate the potential of deep-learning-based virtual staining in 3D X-ray histology, we apply our methodology to biodegradable magnesium-based implants in bone tissue. This is a challenging biomaterials system previously examined in multiple correlative studies of alloy degradation and osseo­integration using a range of electron and X-ray microscopy techniques alongside conventional histology (Krüger *et al.*, 2021 ▸; Sefa *et al.*, 2023 ▸; Iskhakova *et al.*, 2024 ▸). In Krüger *et al.* (2022 ▸), comparisons of bone-implant samples at different healing intervals revealed that magnesium alloys gradually form a stable degradation layer, surrounded by newly formed non-mineralized bone. While histology confirmed the presence of these features, SR-µCT combined with a U-Net convolutional neural network (Baltruschat *et al.*, 2021 ▸) enabled tomographic segmentation, allowing 3D visualization and volume-based quantification of key parameters such as bone-implant contact and degradation rate. Although corresponding 2D histology and 3D µCT measurements were correlated, discrepancies in detail highlight this system as a strong potential candidate for 3D virtual staining. This poses the primary question of whether virtual staining can accurately infer stain-specific contrast from greyscale µCT volumes, thus enabling virtual sectioning of the implant and surrounding tissue in arbitrary orientations without additional labelling, sectioning, tissue clearing, or decalcification. For this investigation, to facilitate supervised learning, we collated and co-registered paired datasets from such prior studies, comprising upwards of 50 SR-µCT slices and their corresponding stained histological sections. An exemplary pair is shown in Fig. 1 ▸, annotated with key sample features referenced in the text.

## 2. Methods and materials

Collected imaging datasets from the aforementioned correlative characterization studies of bone-implant systems were re-used in this computational project, pertaining to samples with explants of magnesium–gadolinium screws (Mg–5wt%Gd and Mg–10wt%Gd), titanium (Ti) or polyether-ether-kethone (PEEK) screws implanted into Sprague Dawley rat tibia with healing periods of 4, 8 or 12 weeks. The Ti and PEEK screws were used as control materials to evaluate osseointegration without degradation of the implant. Animal experiments were conducted after ethical approval by the ethical committee at the Malmo/Lund regional board for animal research, Swedish Board of Agriculture (approval number DNR M 188-15). For a comprehensive description of the sample preparation methodology which includes the initial alloy production and animal study details, refer to Krüger *et al.* (2022 ▸) and references therein.

**Figure 2:**
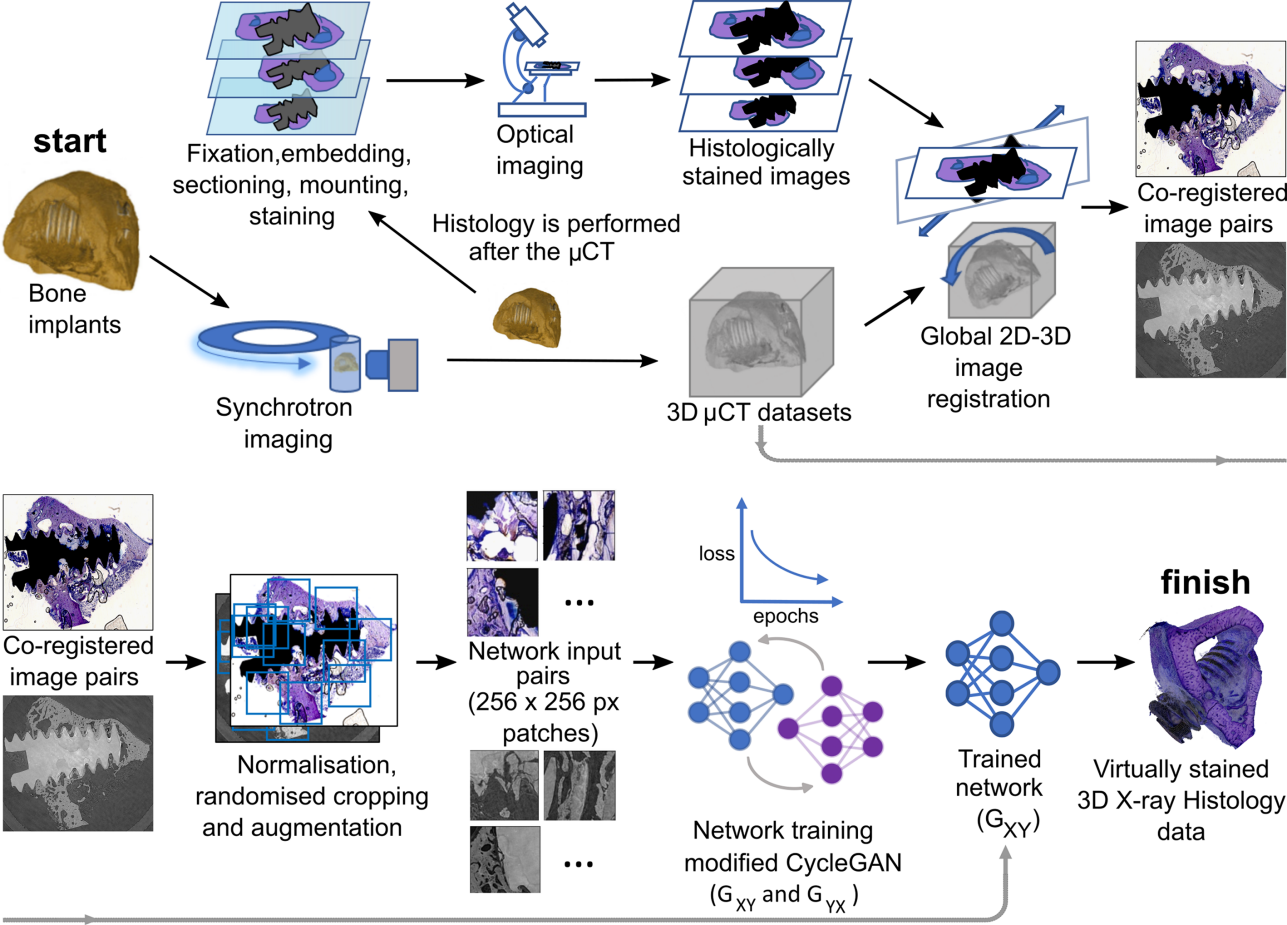
Schematic of the methodology for 3D virtual staining in X-ray histology of bone implants, using a paired CycleGAN network.

Following the sample preparation of the bone-implant block, the methodology timeline is illustrated schematically in Fig. 2 ▸ which comprises the two imaging modalities followed by global registration to create the image pairs required for the supervised GAN inputs. Note that the histology must be performed after the tomography due to the destructive nature of the histological sectioning process. The second half of the methodology describes the investigated models, the data and its treatment including normalization, augmentation and use of a sample correspondence mask, as well as the metrics applied for comparative 2D analysis. Finally, we demonstrate the trained model in a 3D application by processing a stack of µCT slices into a virtually stained 3D X-ray histology volume.

### 2.1. Sample preparation

For the SR-µCT imaging, samples consisted of bone-implant blocks comprising screw (4 mm length, 2 mm in diameter and M2 thread) and surrounding bone tissue, explanted with a trephine bur of 6 mm diameter. The bone-implant blocks were fixed in 70% ethanol for one or more days and then dehydrated in a graded series of ethanol (samples were critically point dried). The dried blocks were put into a standard Eppendorf tube (with arbitrary orientation) before being placed in the SR X-ray beam for tomographic imaging.

For the histology, which occurred after the SR-µCT imaging, explants were re-infiltrated with absolute ethanol and then embedded in methyl methacrylate resin by LLS Rowiak LaserLabSolutions GmbH (Hanover, Germany). Each sample was cut in half along the screw longitudinal axis with an Exakt saw, and then prepared for non-decalcified histology with the cutting-grinding technique *ad modum* Donath (1988 ▸). Sections of about 40 µm were obtained, mounted on glass slides and stained (Histlab, Göteborg, Sweden) with a solution of toluidine-blue/pyronine-Y. Toluidine blue solution is widely used as a staining method for undecalcified bone tissue, allowing for identification of the mineralized bone matrix, osteoids and soft tissues (Peev *et al.*, 2024 ▸). When combined with pyronin Y it results in staining of the bone tissue in various shades of purple (darker corresponding to younger bone) while the soft tissue is stained with more of a blue tint (Sarve *et al.*, 2007 ▸). New woven bone at the degradation layer interface may also be stained bright blue (Krüger *et al.*, 2022 ▸).

As a smaller secondary dataset, we also collated histology samples that were stained with hematoxylin and eosin (H&E) (LLS Rowiak, Hannover Germany). Sections were laser-cut by LLS Rowiak in a process which excludes the residual screw implant from the final mounted tissue sections (whilst retaining a portion of the screw degradation layer material). H&E is the most widely used stain across all cell types; in bone it results in a deep pink, almost red stain for the mineralized tissue and purple staining of the soft tissue. Examples of this less complete dataset are presented in Section S2 of the supporting information.

### 2.2. Imaging

The SR-µCT datasets were acquired over several beam times at the imaging beamline (IBL) P05, which is operated by the Helmholtz-Zentrum Hereon, at the PETRA III storage ring of the Deutsches Elektronen Synchrotron (DESY) in Hamburg, Germany (Wilde *et al.*, 2016 ▸). The primary form of contrast is attenuation-based, with minimal in-line phase contrast, acquired in full-field transmission geometry. As the beam times were part of different studies, a range of various acquisition parameters were utilized, including X-ray energies (ranging from 25 to 45 keV to accommodate the attenuation range of the different screw alloy materials, *i.e.*, PEEK, Mg and Ti) and cameras (CCD versus CMOS) coupled to the X-ray microscope. Further details are given by Krüger *et al.* (2022 ▸). Volumes of approximately 6 mm × 6 mm × 6 mm were scanned, with effective pixel sizes of either 1.2 or 2.4 µm. Tomograms were reconstructed using a MATLAB-based framework and via *ASTRA toolbox* for tomographic backprojection. Following tomographic reconstruction all datasets were downsampled to a voxel size of 5 µm (1.5k × 1.5k pixel slices).

The collected histological images were also obtained over multiple measurement periods. All stained sections were imaged with one of two white-light optical microscopes coupled to a camera (Optical microscope 1: Nikon Eclipse Ci-L and DS-Fi3 camera controlled by NIS-Elements software, Tokyo, Japan; Optical microscope 2: Zeiss AxioCam MrC controlled by AxioVision software, Oberkochen, Germany). Images were acquired with nominal magnifications of 5×, 10× or 20× in wide field mode (for effective pixel sizes of 0.88 µm, 0.44 µm or 0.22 µm, respectively), and whole slide imaging (WSI) was achieved via guided manual stepping of the sample stage together with the inbuilt automatic image tiling function. Some sample images may be described as a partial WSI centred on the screw (approximate field of view is 2.5 mm × 4.5 mm), while others contain the full extent of sectioned screw plus bone area (roughly 6 mm × 6 mm). There is no consistent final array size with typical dimensions ranging from 6k to 13k pixels. Following registration (described in the next section), the transformed WSI images were downscaled to match the pixel size of the µCT slice pair (5 µm).

### 2.3. Registration

SR-µCT and histology WSI image pairs were co-registered using a semi-automated global 2D–3D registration pipeline that has been custom-written in Python, outlined by Irvine *et al.* (2024 ▸). In an iterative process based on optimizing the mutual information metric, a 3D rigid transformation is applied to the µCT dataset to find the optimal virtual plane fit to the histology image. This is followed by an affine transformation of the histology image, incorporating non-uniform scaling and local shear to compensate for minor distortions introduced during histological sample preparation after the tomography measurements. Due to the rigidity of the mineralized bone, deformation components were small, with shear parameters of <1% for the toluidine blue-stained samples and up to 3–4% for the H&E-stained samples. The pixel size of the transformed output registered histology/µCT image pair is the same as the input µCT dataset. Finally, both transformed slices were cropped to fit the minimum field of view of the pair.

### 2.4. Datasets

A total of 53 co-registered µCT and toluidine-blue stained histology WSI pairs were collated for this project. These comprised 38 samples containing Mg (-based implants), five containing Ti and ten containing PEEK. We performed a five-fold cross-validation with a 40/10 train/validation split rotated through five times. Each validation set was randomly selected whilst keeping a representative 7:1:2 ratio of Mg:Ti:PEEK samples. The three WSI pairs for testing (comprising two Mg and one PEEK) were kept separate and never involved in training in order to prevent data leakage.

Corresponding details of the secondary H&E-stained dataset are given in Section S2.1 of the supporting information.

### 2.5. Normalization

Normalization was applied to all histology and µCT image pairs to mitigate domain shift. As the data were compiled from multiple measurement sessions and datasets, they exhibit substantial variation in image quality and statistics. For both modalities, regions of interest (ROIs) of both rigid bone and air/background were sampled to define scaling bounds. With the µCT data, intensities were linearly scaled to set the mean values of bone and background (30000 and 10000, respectively, for a 16-bit greyscale image file). For the histology RGB images, a simple white-balance correction was applied via the mean sampled background value μ_R,G,B_ (such that μ_R_ = μ_G_ = μ_B_), followed by contrast-stretching. Distribution bounds were defined by the first percentile of the bone ROI intensity distribution *i* (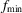 = 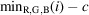, clamped to 10) and the 99th percentile of the background ROI distribution *j* (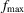 = 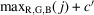, clamped to 255 for 8-bit images). The same constants *c* and *c*′ were applied across all channels to preserve chromaticity.

### 2.6. Network models

#### 2.6.1. CycleGAN and its adaptation for paired data

**Figure 3:**
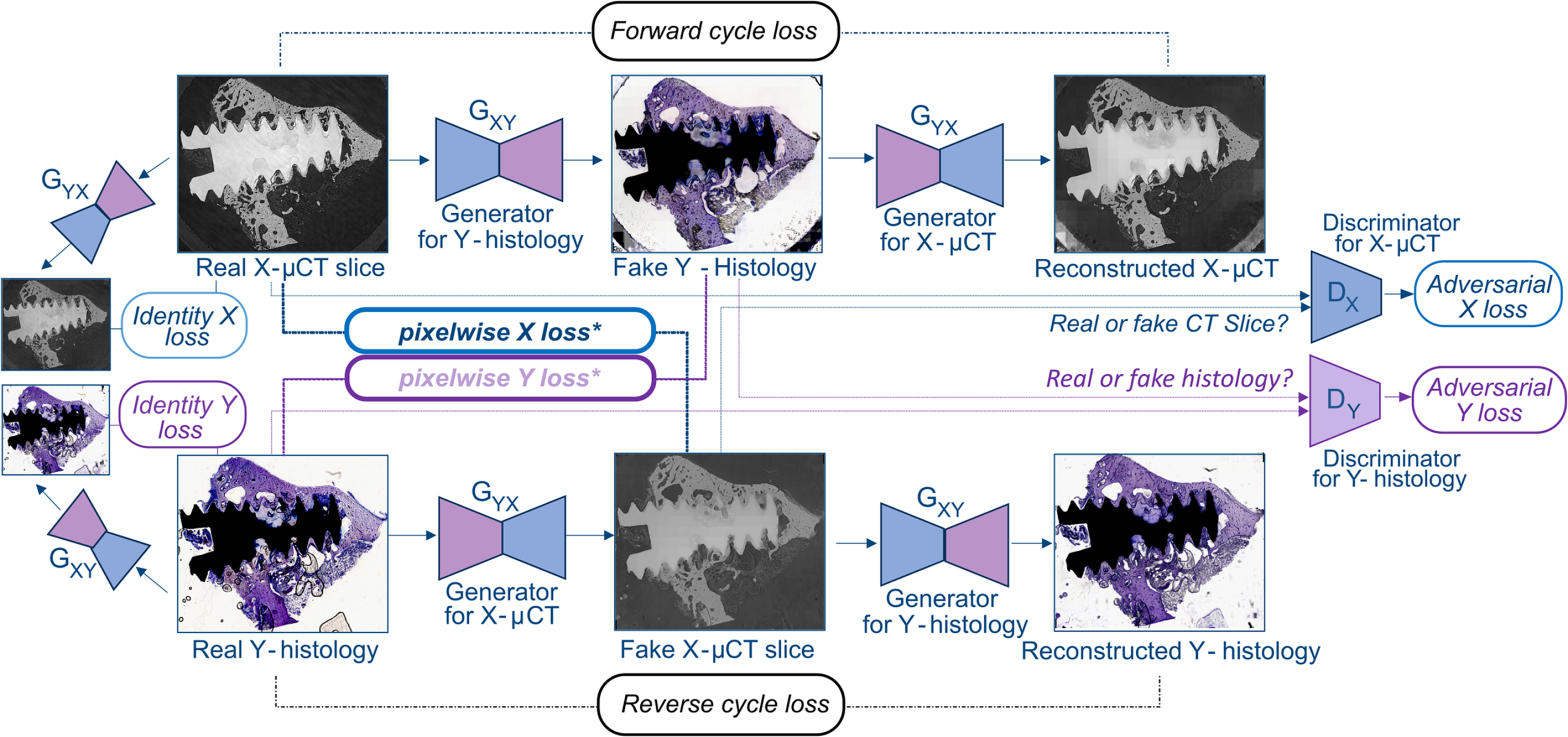
The modified CycleGAN for paired data, with additional pixelwise supervision loss terms that directly penalise differences between the generator output and the target image for each domain. Not shown: an additional greyscale loss term applicable to the generated µCT data only.

Our chosen model is based on the CycleGAN framework and adapted for paired data. We worked with a *PyTorch* implementation of the original CycleGAN model (Zhu *et al.*, 2017 ▸) as developed by Linder-Norén (2019 ▸). A schematic of our modified model is illustrated in Fig. 3 ▸.

The base model employs two generator networks: *G*_*XY*_, which learns to translate images from the source domain *X* (comprising µCT scans) to the target domain *Y* (histology images), and *G*_*YX*_, which performs the inverse mapping. Each generator is trained adversarially against a corresponding discriminator (*D*_*Y*_ and *D*_*X*_), which learns to distinguish real from generated images.

Since in the original case no direct correspondence was assumed to exist between images in the two domains, the cycle-consistency constraint is introduced to ensure that an input image can be approximately recovered after translation and back-translation. Specifically, given an image *x* ∈ *X*, the composition *G*_*YX*_[*G*_*XY*_(*x*)] should closely match the original *x*. This acts as a form of self-supervision, allowing the network to learn meaningful mappings even without paired data. The cycle-consistency loss is defined as
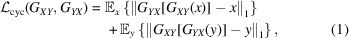
whereby an ℓ_1_ (or mean absolute error) loss is applied between the original images and the reconstructed images, and 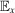 and 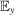 denote expectations over the respective data distributions.

For the adversarial loss: we use a mean squared error, or ℓ_2_, loss function between discriminator predictions and real/fake labels. The generator adversarial loss is given by
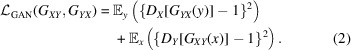
An additional identity loss encourages preservation of colour and content when mapping images already in the target domain, of the form
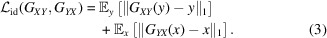
Equations (1), (2) and (3) represent the three loss functions commonly comprising the standard CycleGAN model. The total generative loss function for the original CycleGAN model combines these three components, weighted by hyperparameters λ_cyc_ and λ_id_,
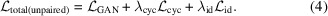
For our modified CycleGAN we adapt this model to paired data, whereby each image *x* ∈ *X* is assumed to have a corresponding ground truth image *y* ∈ *Y*, and vice versa. To take advantage of this, we introduce a ‘pixelwise supervision loss’ that directly penalizes differences between the generator output 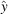 = 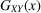 and the target image *y*, as well as 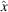 = 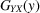 and the target image *x*. This is formulated as a combined ℓ_1_ loss,
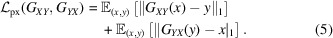
Lastly, we introduce a loss term which helps ensure that the generated µCT image is purely greyscale,
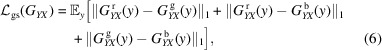
where 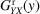, 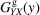 and 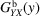 are the red, blue and green channels of the generated µCT image. (To present as a greyscale image, all three channel values should be equivalent.)

The total generative loss for our paired datasets combines all components, weighted by hyperparameters λ_cyc_ and λ_id_, λ_px_ and λ_gs_, 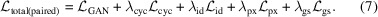
In this study, we evaluate the performance of the CycleGAN network as applied to paired data, using both the standard total generator loss [equation (4)] and our modified version [equation (7)].

#### 2.6.2. Pix2Pix

As a further baseline comparison, we test our modified CycleGAN for paired data against the classic Pix2Pix model (Isola *et al.*, 2017 ▸), which is a conditional generative adversarial network (cGAN). In this framework, the generator *G* learns to map an input image *x* (here a µCT slice) to a corresponding target image *y* (histological section), *i.e.G*(*x*) ≃ *y*. The discriminator *D* is explicitly conditioned on the input *x* and is trained to distinguish between real image pairs (*x*, *y*) and fake pairs [*x*, *G*(*x*)].

The conditional adversarial loss between generator and discriminator can again be written as an ℓ_2_ loss,
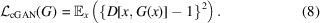
This adversarial loss is balanced in the Pix2Pix model with a pixelwise loss commonly referred to the ℓ_1_ loss,
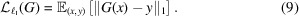
The total generative loss for Pix2pix then combines them with the weighted parameter 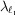,
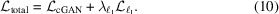


#### 2.6.3. Model architectures and hyperparameters

The base model architecture for both the CycleGAN model variations and Pix2Pix models tested follow the *PyTorch* implementations of Linder-Norén (2019 ▸). All models were initialized from scratch without pre-training before introducing our input data.

For the CycleGAN model variations, the generator network is based on a ResNet backbone with nine residual blocks and a starting feature width of 64. The discriminator follows an unconditional PatchGAN design with a 70 × 70 pixel receptive field. To stabilize adversarial training, an image buffer was used to store previously generated samples for the discriminator loss, following the technique described by Zhu *et al.* (2017 ▸). Training was conducted using the Adam optimiser with a learning rate of *l*_*r*_ = 0.0002, and momentum parameters β_1_ = 0.5 and β_2_ = 0.999. For our modified CycleGAN loss model for paired data given in equation (7), we achieved good results with loss factors λ_cyc_ = 6, λ_id_ = 3, λ_px_ = 6 and λ_gs_ = 1. These factors are approximately proportional to those relative to the GAN loss as optimized in the original study by Zhu *et al.* (2017 ▸). The latter two factors are effectively ablated for the comparison with the standard CycleGAN model given by equation (4). Sample correspondence masks (see Section 2.8) were applied as binary multipliers for all ℓ_1_ loss terms.

For the Pix2Pix model, the standard architecture as introduced by Isola *et al.* (2017 ▸) consists of a U-Net generator and a conditional PatchGAN discriminator. Here the total loss model follows equation (10), where 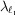 = 20 after tuning. We also applied the sample correspondence mask to the ℓ_1_ pixel loss term. All other architectural and training settings were kept consistent with those used in the CycleGAN model above.

In each case models were trained on 256 × 256 pixel patches randomly sampled from 40 WSI pairs, with 100 patches per pair per epoch, a batch size of four, and a total of 500 epochs. At each epoch, new randomized data augmentations were applied on-the-fly during patch extraction. During training, model states corresponding to the minima of each individual generator loss component were saved as checkpoints for later evaluation. Each training fold required approximately 100 h on an NVIDIA Tesla V100 GPU for the CycleGAN models versus 40 h for Pix2Pix, across the fivefold cross-validation scheme.

### 2.7. Data augmentation

The utility of data augmentation in achieving learning invariance has been well demonstrated (Dosovitskiy *et al.*, 2014 ▸; Ronneberger *et al.*, 2015 ▸) and is particularly beneficial in medical imaging applications, where obtaining large numbers of paired datasets is often a challenge. By combining the randomized patch-based sampling with further data augmentation at each epoch we were able to simulate a substantially larger paired dataset than the initial set of WSI image pairs, thereby helping to prevent overfitting. For each training example in both histology and µCT images, a random crop position was determined for the sampled patch. We also incorporated rescaling by 90–110% (whilst maintaining patch array size), horizontal and vertical flipping, and a separate contrast and brightness jitter (up to 10%) for both the µCT and histology patches. For the latter, the chromaticity or perceived colour is not varied (RGB ratios are preserved).

### 2.8. Sample correspondence

Sample correspondence masks were applied to the ℓ_1_ loss terms during training in order to exclude regions of the image pairs where there was known to be a mismatch between µCT and histology. These could be either in the imaging field of view or physical differences in the sample between tomographic and histological measurement arising from the sectioning process. The maps were created using a convex hull mask of the rigid bone tissue+screw sample, calculated via intensity-based segmentation. This method includes any soft tissue which is surrounded by the rigid bone material which is assumed to remain part of the sectioning, as well as a small amount of background pixels surrounding the bone. This effectively excludes from the training a majority of extraneous sample material (mainly soft tissue) in the µCT volume which did not end up in the histology section, as well as the walls of the sample holder which contained the bone-implant block. These walls can be seen in many of the CT slices. We generated convex hull masks for both CT and histology and took the minimum union, combined with a third/fourth mask generated with semi-manual selection for regions of any missing other information not accurately defined by the convex hull mask (*e.g.* histology images with missing sections of bone due to irregular sectioning). In general we did not attempt to mask out any small imperfections in the histology images, such as cracks in the optical slides, stain droplets, *etc*. The masks were also subsequently applied to our showcased results and during their comparative analysis, whereby any pixels outside of the sample correspondence mask were replaced with a value equivalent to a mean background value.

### 2.9. Whole slide image outputs

Within the field of virtual staining (with visible light), most methodological studies are constrained to patch-level analysis due to the gigapixel scale of WSIs at their original resolution (Liu *et al.*, 2025 ▸). Since we work with downscaled WSIs at the resolution of the input µCT data, we do not have the same memory-related constraints. Following the patch-based training of our model, we re-tiled the model outputs to obtain overlapping patch-based inference of whole slide images of the order 1k × 1k pixels. Analysis of model performance across the WSI field of view has several advantages over a patch-level analysis. This approach provides a more comprehensive assessment of output quality, as localized patches may appear visually convincing in isolation (passing the visual Turing test) but fail to reflect broader inconsistencies. In contrast, overlapping patch-based WSIs may reveal global artefacts such as tiling borders or inconsistent transitions that arise in cases of model instability. Although direct WSI inference is also possible given network scalability, we nevertheless retain patch-based inference for consistency with training and to preserve the same spatial frequency sampling.

### 2.10. Comparative metrics

In addition to a visual assessment of each model’s performance, we also applied the following complementary metrics for a comprehensive quantitative comparison: structural similarity index measure (SSIM), peak signal-to-noise ratio (PSNR) and learned perceptual image patch similarity (LPIPS). These metrics capture different aspects of image similarity, including structural coherence, pixel-wise differences and perceptual fidelity.

We applied the metrics through patch-based (256 × 256 pixels) sampling of the inferred WSI histology images with reference to their input histology counterparts. Background patches were excluded from analysis according to the sample correspondence mask (defined by a 50% overlap or greater).

The structural similarity index measure, or SSIM (Wang *et al.*, 2004 ▸), quantifies perceptual similarity between two images based on luminance, contrast and structural components. It is defined as
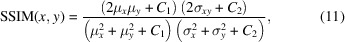
where μ_*x*_ and μ_*y*_ are the mean intensities, 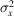 and 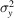 are the variances, σ_*xy*_ is the covariance, and *C*_1_, *C*_2_ are small stability constants. We used the SSIM implementation by Van der Walt *et al.* (2014 ▸).

The PSNR is a traditional metric for quantifying absolute error of the reconstruction quality (between real and generated images) on a logarithmic scale,
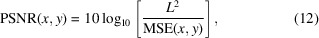
where *L* is the maximum possible pixel value (*i.e.* 255 for 8-bit images) and MSE(*x*, *y*) is the mean squared error between the images, 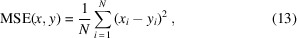
where *N* is the number of pixels.

The learned perceptual image patch similarity, or LPIPS (Zhang *et al.*, 2018 ▸), measures perceptual similarity by comparing deep features extracted from a pretrained neural network. Unlike SSIM or PSNR, LPIPS captures high-level perceptual and colour differences, making it well suited for evaluating visual fidelity in generative tasks in the RGB space. Given feature maps *f*_*l*_(*x*) and *f*_*l*_(*y*) at layer *l*, the LPIPS distance is
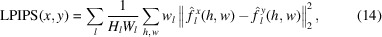
where 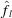 denotes channel-wise normalized feature maps and *w*_*l*_ are learned weights. We used the LPIPS implementation by Detlefsen *et al.* (2022 ▸) with the SqueezeNet network type.

### 2.11. 3D testing

Once our chosen network model was trained, we applied the forward generative model to a stack of input SR-µCT slices for validation in 3D. These were untransformed slices, although one training pair was also produced from this dataset through the co-registration process outlined in Section 2.3. A 3D Gaussian filter with a σ of one pixel was first applied to the CT slice stack, in order to simulate the resolution reduction which occurs through interpolation steps of the co-registration process. The output stained slices were generated from the trained forward model independently as single slices. For 3D visualization these slices were loaded into Avizo3D 2024.2 (Thermo Fisher Scientific, Berlin, Germany), and the inverted mean of the RGB channels was computed as the alpha (A) channel. This enabled a volume rendering with direct RGBA mapping.

## 3. Results and discussion

**Figure 4:**
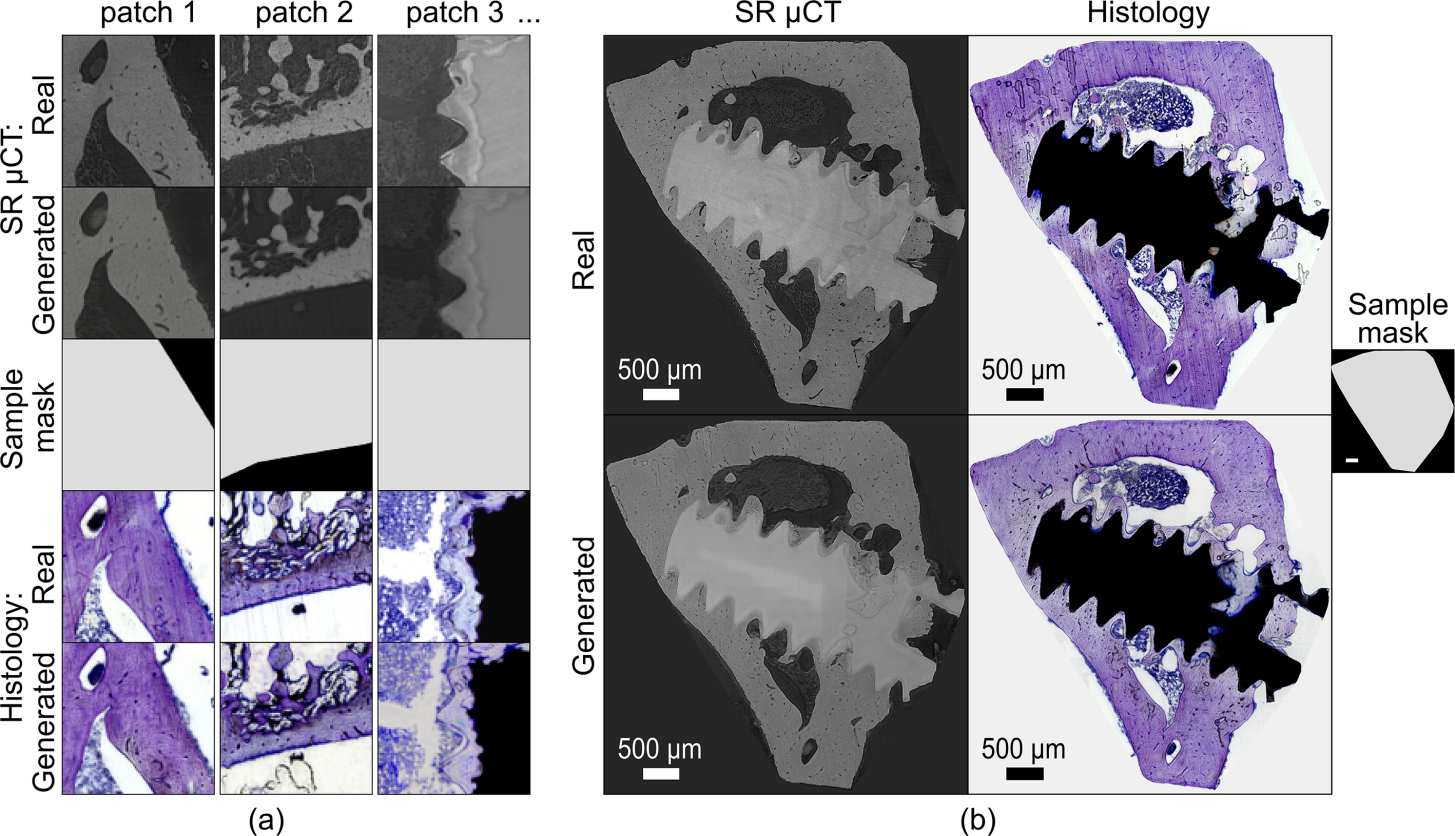
Examples of our modified CycleGAN model training results: (*a*) direct training patch input/outputs (256 × 256 pixels); and (*b*) example WSI result generated through overlapping patch-based inference, from one WSI sample pair included in training (958 × 1000 pixels). The displayed µCT and histology images have been masked (replacing all background pixels with the mean value) with the included sample correspondence mask.

An example of the direct training patch results of our modified CycleGAN model is shown in Fig. 4 ▸(*a*). For each 256 × 256 pixel patch region, there is the input real SR-µCT patch, and output generated histology patch, as well as the input real histology patch and corresponding output generated µCT patch. In addition, the sample correspondence map patch is also shown in the central panel. At the patch level, the similarity between input and generated images appears very high. However, it is possible to see that the patches are not perfectly aligned down to every pixel. This is partly due to the initial registration, which was performed solely on a global scale of the WSI and not subsequently re-applied to each individual patch, as well as to differences in the sample slice pair which arise from the histological sectioning process. In particular, the soft bone tissues [as shown in blue to the left of patch 3 of Fig. 4 ▸(*a*)] were observed to shift and these regions are not co-registered as accurately as the rigid bone structures. In Fig. 4 ▸(*b*), a representative WSI training result is shown, as generated through overlapping patch-based inference (stepping the 256 × 256 pixel patch across with a step size of 64 pixels). The sample correspondence mask (also shown separately) has been applied after image generation whereby pixels exterior to the sample are set to the mean background value. Examples of the inferred WSI training, validation and test results displayed without the mask are given in Fig. S1 of the supporting information. Behind the mask, the effect of overlapping patches can sometimes be observed in background regions of the generated images in the form of tiling artefacts of varying intensity steps, reflecting uncertainty in the model prediction due to exclusion from the training process. The step size may be reduced to smooth this effect; however, generally in well trained image regions there are no such artefacts. Note that, for the remainder of this manuscript, generated image results will be presented as masked overlapping patch-based inferred WSI images, or a region of interest thereof, and not the original patches directly used as model input/output.

**Figure 5:**
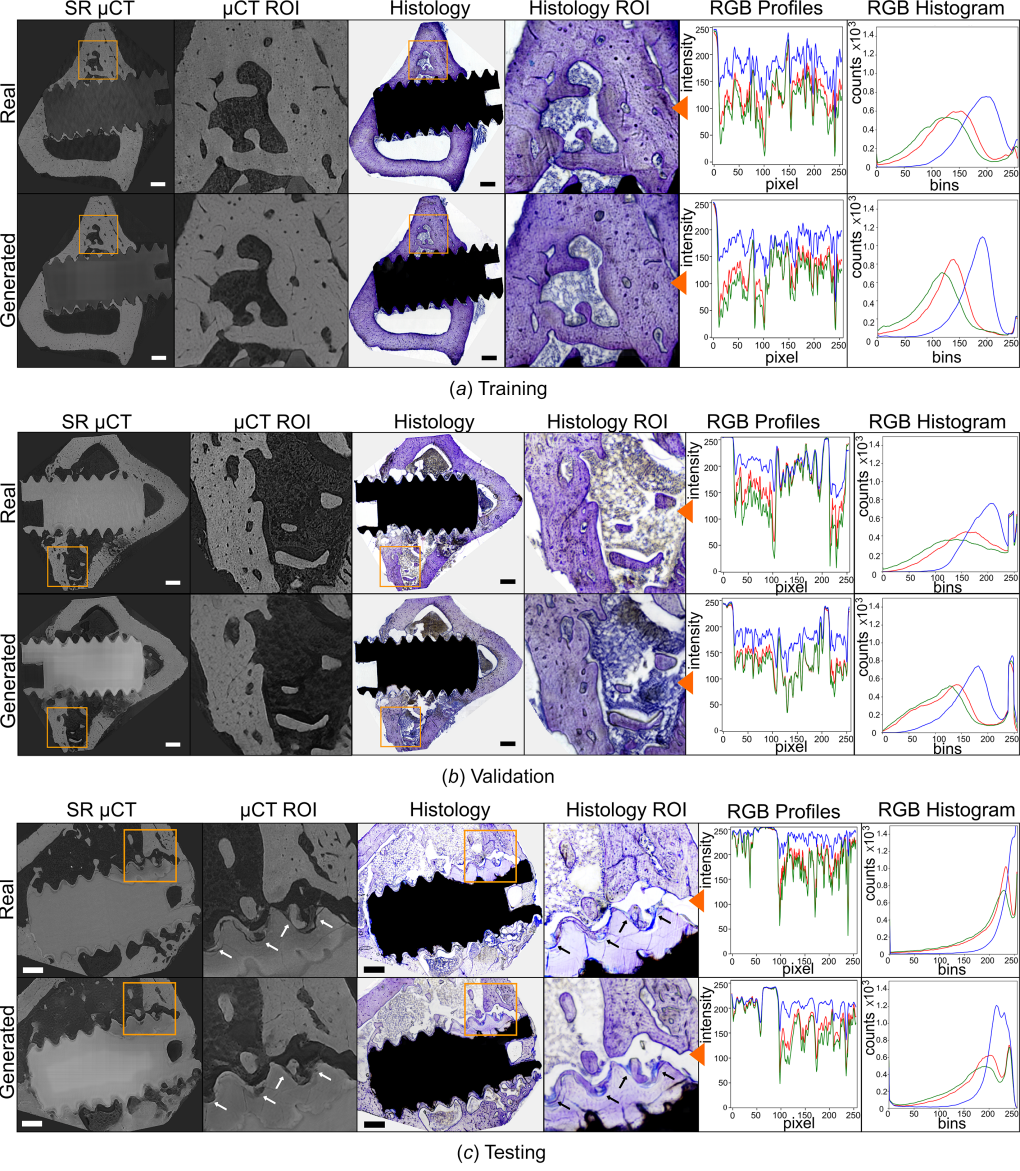
Example modified CycleGAN model results (with WSI output) for (*a*) training, (*b*) validation and (*c*) testing. Includes a 256 × 256 pixels region of interest (ROI) with accompanying RGB histograms and intensity profiles of real versus generated examples. Each profile is acquired horizontally across the midpoint of the ROI as indicated by the orange arrow. The scalebar in each WSI represents 500 µm. In (*c*), isolated regions of new bone growth are indicated with arrows in white (µCT) and black (histology), for both the input data and generated histology.

Representative results of our modified CycleGAN model from training, validation and testing datasets are demonstrated in Fig. 5 ▸. Overall there is a good apparent level of agreement between real and generated WSIs in both domains for each dataset, although, as to be expected, there is a notable drop in perceived match accuracy when comparing against the training results. In particular, the generated WSI histology training results appear excellently matched.

In general, the forward model (SR-µCT to predicted histology) results of the CycleGAN are more likely to pass a visual Turing test than the reverse model (histology to predicted SR-µCT). Within the generated µCT slices, tiling artefacts (as mentioned above) are present in the majority of regions containing the residual screw alloy (represented in black pixels within the corresponding histology). Unlike the background regions, these screw regions were not excluded from the training process by the sample correspondence masks. However, the uncertainty in their prediction lies in the possibility of three different material components (PEEK, Mg or Ti) within the sample pool which are characterized by three different linear attenuation coefficients for SR-µCT (respectively, less than, approximately equal to, or greater than the value for dense bone tissue). Each of these are matched to the same black values of the histology input. In our preliminary studies previously reported by Irvine *et al.* (2024 ▸), we initially incorporated a fourth channel (in addition to R, G and B channels) as model input in which the known screw material could be indicated as a single value. However, this extra parameter was eventually found to contribute to a significant blurring effect over the whole generated image and we have since abandoned this in our current model. Further contributing to their synthetic appearance, the generated CT slices tend to exhibit reduced high-frequency detail compared with their real counterparts, and lack the shot noise characteristic of real X-ray images.

Other noted discrepancies between real and generated images include the many characteristic features of the real histology sample images which have not been replicated in the generated histology. These include cracks in bone, saw marks (*i.e.* one-directional striations), histology slide fractures and stain droplet spills. The same histology features were mostly reproduced in greyscale format within the generated CT slices, also distinguishing them from their real CT counterpart. All of these features were generally included in the training (unless located significantly external to the bone sample in which case they were masked out); however, they were not able to be ‘learned’ as features due to a lack of correspondence. These features are not intrinsically related to the sample but are formed as part of the histological sample preparation process, and are absent from the CT acquisition. This highlights the distinction between accuracy, referring to faithful structural representation, and realism, which encompasses visual artefacts that enhance stylistic plausibility. The omission of such features reflects the model’s preference for preserving structural accuracy over superficial realism.

The colour, contrast and resolution fidelity of our paired CycleGAN output results are demonstrated in more detail with the selected ROIs and corresponding RGB histogram plots and intensity profiles derived from the histology ROIs, within Fig. 5 ▸. As expected, the training results are a superior match to the validation and test results, most notably with respect to the colour fidelity of the generated histology, but also in the reproduction of the finest features. In the training data (Fig. 5 ▸), both real µCT and histology ROIs exhibit a large number of medium and small-sized pores visible down to only a few pixels in size within the dense bone structure, which correspond to the blood vessels and lacunae, respectively. These pores are well reproduced in the generated histology, demonstrating the high level of detail able to be supported by the forward model. However, some fine linear features visible in the real histology and correspondingly in the generated µCT images are absent from the generated histology. These likely represent vessels or canaliculi oriented parallel to the cutting plane, indicating a discrepancy between the thickness of the histology sections and registered virtual µCT slices which could be better matched for the model training. Accordingly, the histograms and profiles of the training set are very similar although non-identical. In the validation data set [Fig. 5 ▸(*b*)], the soft tissue in the generated histology is notably of a different colour in certain areas to the real image (with respect to blue and brown), as well as differing in intensity, although the variations could be considered realistic as they reflect the varying colours and intensities of soft tissues observed elsewhere in the histology dataset. Such differences are reflected in the histogram, and in the RGB ratios at levels attributable to the soft tissue pixels. The lacunae and other fine features are also less visible in comparison with the training example. In the test example (Fig. 5 ▸), discrepancies between real and generated histologies in the colour of both dense bone and soft tissue may also be observed. Conversely to the validation ROI above, the generated soft tissue here is skewed more towards brown than the blue of the real soft tissue. Additionally, we note that our test input µCT data are of lower resolution than the typical 50 training and validation CT examples. As a result, the generated histology also contains fewer fine features, which is visible in broadened histogram peaks and smoother line profile. However, the loss of detail is not as pronounced as one might expect based solely on the input CT resolution, particularly in the dense bone regions. In fact, while a comprehensive spatial frequency model analysis is beyond the scope of this study, preliminary findings (see Section S3) suggest that the GAN-generated histology typically gains a spatial resolution that is lower than real histology but higher than the input µCT. This places the output resolution between the two modalities and supports the qualitative observation that fine structural details may be partially preserved, despite the lower-resolution input.

As a final qualitative test, the selected testing ROI in Fig. 5 ▸ provides a valuable demonstration of the GAN generator’s predictive accuracy in capturing certain key features from the original X-ray histology study, namely the Mg alloy screw degradation layer and potential regions of new (woven) bone growth (see also Fig. 1 ▸ for reference). Here we observe that the prediction for the degradation layer exhibits a boundary that is reasonably consistent with the real histology although a blurriness at the edge of the black residual screw suggests some uncertainty. Upon closer inspection within the degradation layer, we note that, while in real histology images this area is characteristically devoid of bone cells (Krüger *et al.*, 2022 ▸), inside the generated layer a faintly repeating cell-like structure is apparent. This suggests that some features may be falsely enhanced from image noise, and more representative data of degradation layers are ideally required for the model to train upon. Importantly, the generated model appears to have successfully predicted the presence of new bone, as indicated by the bright blue regions next to the degradation layer (annotated in the ROI by small black arrows) which were matched to areas of reduced density within the CT image (white arrows).

Results of our modified CycleGAN model on the secondary H&E-stained dataset are provided in Section S2. While the dataset is too small for extensive interpretation, these results are consistent with our main findings and suggest that the model may generalize across multiple stains.

### 3.1. Qualitative comparison of models

Here we present a comparative analysis of our modified CycleGAN model [including extra ℓ_1_ pixelwise supervision and greyscale loss terms given in equation (7)] as evaluated against Pix2Pix representing the baseline model for paired data, as well as the standard CycleGAN model, which also receives paired inputs but is trained using only the standard generator loss [equation (4)]. Whilst the first two models both performed in a predictable manner, the standard CycleGAN model was observed to perform poorly and did not converge to a stable equilibrium. As the only model without direct supervisory loss terms, it was unable to overcome the strong intensity mismatch between the CT and histology domains. The forward generator attempted to create histology images with a dark background and light bone structure, whereas the reverse generator tried to create CT images with a bright background and dark bone structure. This issue is consistent with challenges previously reported when applying CycleGAN to virtual staining tasks with unlabeled microscopy images which present an inverted contrast to the desired stained target images (Bai *et al.*, 2023 ▸). To mitigate this, we adopted a simple recommended workaround (Chen *et al.*, 2021 ▸; Abraham *et al.*, 2022 ▸) and performed a second set of tests of the standard CycleGAN model whereby the input CT images were first inverted. This adjustment led to some limited improvement in both output quality and training stability. The following results are thus included for each of four model variants which includes the standard CycleGAN with both original and contrast-inverted CT inputs. For all variants we focus exclusively on the forward output (*i.e.* the generated histology), as this represents the primary objective of the application. Additionally, because Pix2Pix is composed of only one generator, it does not produce a corresponding CT output.

**Figure 6:**
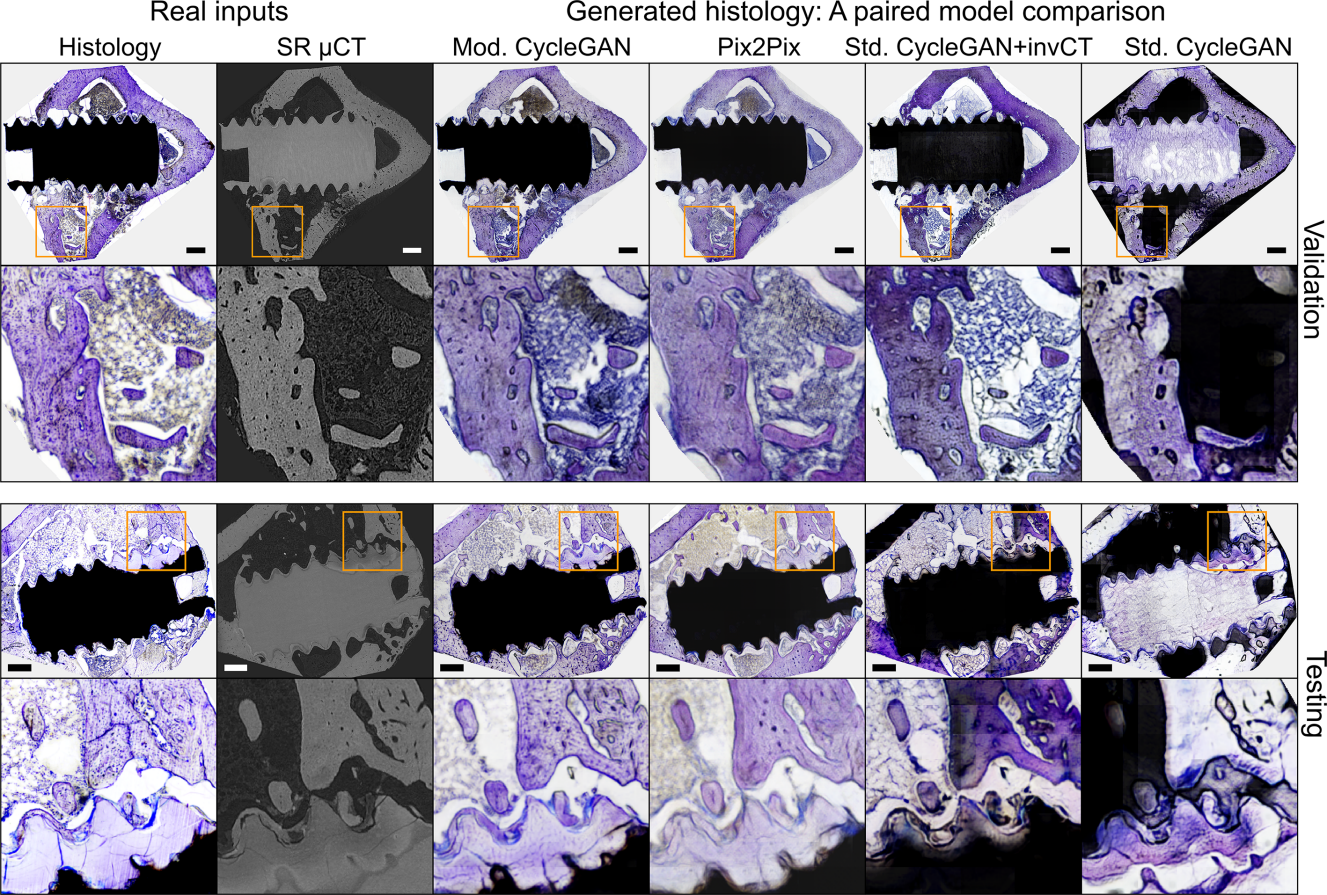
Inference results from each of the paired model variants tested, with WSI and 256 pixel ROI examples from validation and test data sets. ‘Mod. CycleGAN’ refers to the modified CycleGAN including the extra supervisory and greyscale loss terms, which is our chosen model. ‘Std. CycleGAN’ refers to the Standard CycleGAN model, which was trained from the same paired data sets, but without the extra loss terms. ‘Std. CycleGAN + invCT’ refers to a variant of the same model but trained and tested with an inverted CT input, in an attempt to combat the issue of intensity mismatch. The scalebar in each WSI represents 500 µm.

In Fig. 6 ▸ we qualitatively compare the inference results of all paired model variants with example validation and test outputs of a WSI with a 256 × 256 pixel ROI (the same ROI is reused from Fig. 5 ▸). Our model visibly outperforms the competing models. The Pix2Pix model was able to reproduce colour reasonably well; however, the resolution and texture of the generated histology images were noticeably inaccurate. During hyperparameter tuning an emphasis was placed on resolution, which reduced the weight of the ℓ_1_ loss relative to the adversarial term. The resulting increased GAN loss contribution yields some high-frequency artefacts and texture hallucination. The poor performance of Pix2Pix may also be attributed to its sensitivity to the imperfect alignment of paired data, particularly in finer features such as individual bone pores and in soft-tissue regions prone to deformation during histological sample preparation. As mentioned, the standard cycleGAN model (far right) performed very poorly without the additional supervisory loss terms, and using normal CT inputs. The model failed totally to replicate the appropriate histological colours with respect to bone material and background. After switching to an inverted CT input, we observed an increased performance with a more appropriate contrast range, but the model still had trouble with the black screw region of the histology images corresponding to a range of brightness values in the input CT (depending on implant material). This resulted in many regions of bone in the generated histology falsely predicted as black, as well as poor delineation of the degradation layer. Many pores and voids were also predicted with an inverted intensity. Despite a generally poor colour prediction, the standard CycleGAN without extra supervisory ℓ_1_ term was able to reproduce the fine features reasonably well, and in particular the soft tissue regions were more sharply defined than in our chosen model.

Section S2 also includes a reduced model comparison on the H&E-stained dataset, centred upon performance of the modified CycleGAN versus Pix2Pix. The results indicate that the modified CycleGAN maintains an advantage over the Pix2Pix baseline, further supporting its applicability across different staining protocols.

### 3.2. Quantitative evaluation

**Figure 7:**
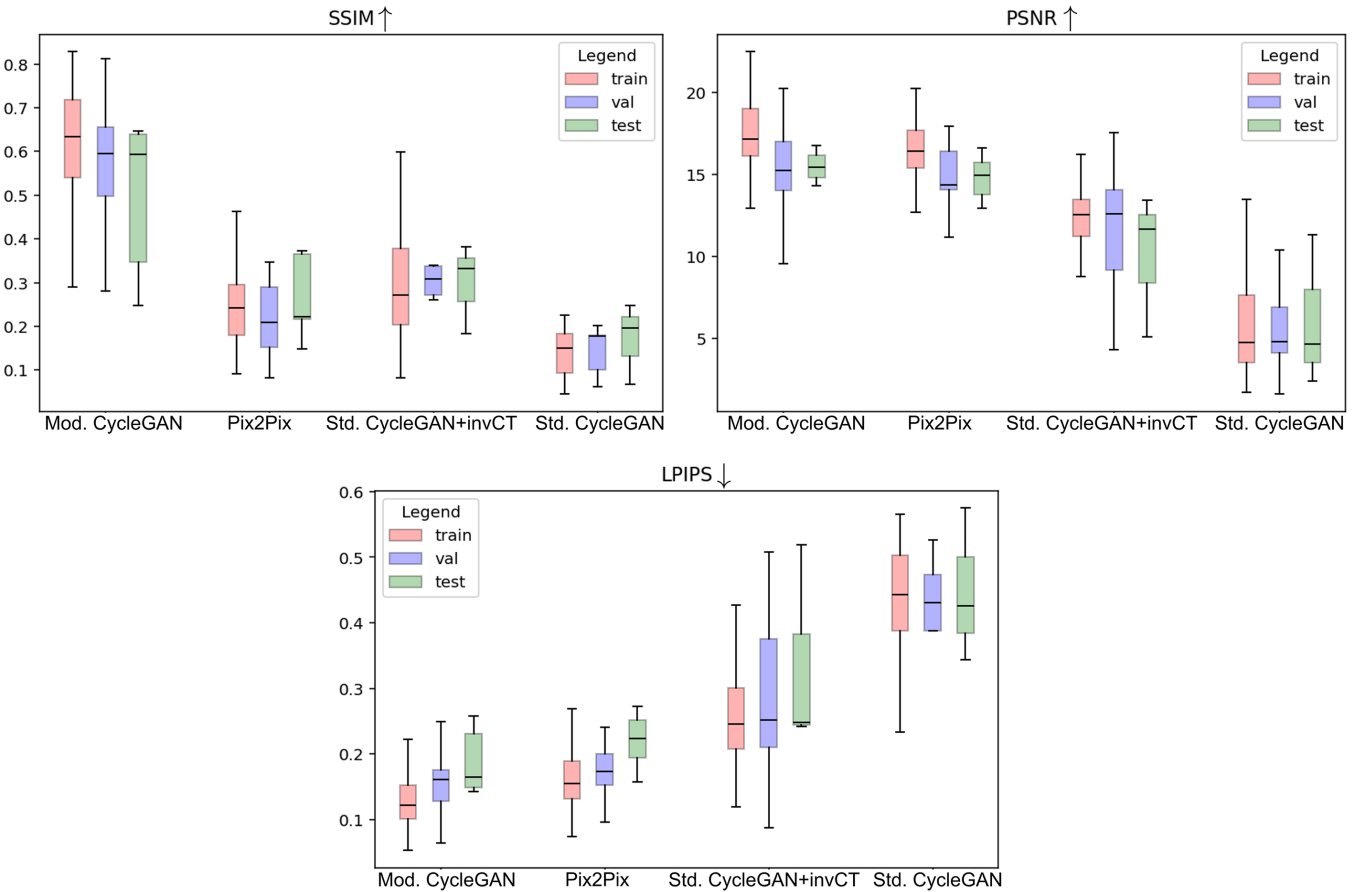
Box plots for a comparison of the paired model variants tested, across the three metrics of structural similarity (SSIM) metric, peak signal-to-noise ratio (PSNR), and learned perceptual image patch similarity (LPIPS).

Table 1 ▸ presents the quantitative measurements of all paired model variants (our modified CycleGAN model with extra loss terms, compared with the standard Pix2Pix and standard CycleGAN models, the latter applied with both normal input µCT and inverted µCT input). Measurement values for each of the metrics SSIM, PSNR and LPIPS are shown, for each of training, validation and testing samples. The values shown are the median calculated values from quite varied distributions due to the highly heterogeneous structures across the sampled WSIs. For this reason we have also included box plots of the distributions in Fig. 7 ▸ where it is possible to observe the range of values.

When evaluated quantitatively, our model consistently outranks all other variants across the three metrics, with particularly strong performance in the structural similarity (SSIM) metric. Among the alternative model variants, the standard CycleGAN with uninverted µCT inputs clearly performed the worst overall. Between Pix2Pix and the standard CycleGAN with inverted µCT inputs, Pix2Pix achieved higher PSNR and lower LPIPS values, indicating lower reconstruction error and a good colour match. However, it performed significantly worse on SSIM, due to the observed high-frequency artefacts. Conversely, the standard CycleGAN with inverted µCT inputs exhibited better SSIM but poor LPIPS, reflecting higher structural fidelity but poor colour consistency.

It is worth noting that while the top scores remain relatively low compared with results from virtual stain transfer, for example, they are comparatively high for other cross-modality image translation tasks. All metric values are somewhat inflated due to the inclusion of screw regions, which comprise large areas of homogeneous black pixels, although these are considered characteristic of our model’s application. As a baseline, when the same metrics were computed between the input µCT slices and their co-registered histology counterparts (*i.e.* without generation), the average SSIM, LPIPS and PSNR were 0.15, 0.4 and 5, respectively. Thus, the standard CycleGAN outputs without inverted CT input are not substantially better matched to the real histology than the raw µCT inputs themselves.

### 3.3. 3D qualitative results from our model

**Figure 8:**
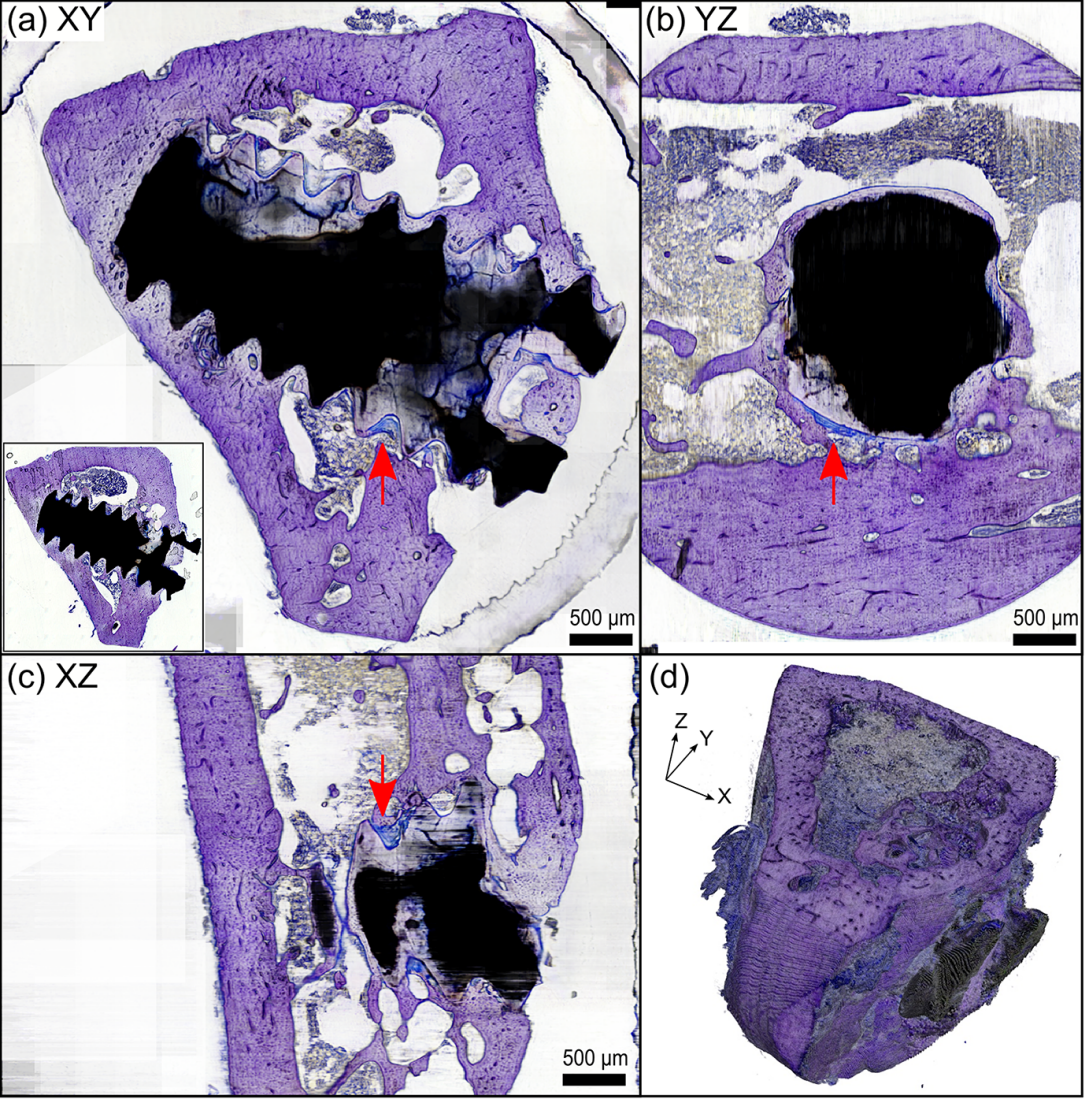
Orthogonal views (*a*, *b*, *c*) and volume rendering with direct RGB mapping (*d*) of a virtually stained 3D X-ray histology dataset featuring an Mg-based degradable bone-implant sample. A real histology image acquired from the same bone-implant sample is shown for reference in the bottom-left inset of (*a*). Red arrows point to a region of new bone growth adjacent to the degradation layer, distinctly coloured in a bright blue.

Orthogonal slice views and a volume rendering of a virtually stained 3D X-ray histology dataset (featuring an Mg-based bone-implant) generated from our trained modified CycleGAN network are shown in Fig. 8 ▸. Overall, the generated volume displays strong visual consistency throughout. However, minor stripe artefacts are visible in the *YZ* and *XZ* planes [Figs. 8(*b*) ▸ and 8(*c*)], caused by slight intensity variations along the slice stack (*Z*) axis. These artefacts are more pronounced in regions containing the degradation layer, where the model shows increased slice-by-slice intensity variation due to uncertainty in predicting the precise interface between the residual alloy and the degradation material. Increasing the size of the training dataset would likely improve performance in this area. Alternatively, generating the volume using multiple orthogonal input planes and combining the outputs [similar to the multi-axes prediction fusing approach outlined by Baltruschat *et al.* (2021 ▸)] should help produce a smoother and more coherent result. In this initial study, we opted for a simpler approach based on independently generated slices, as this best illustrates the model’s stability across the volume.

Notably, our model accurately predicts one of the key regions of interest within the original X-ray histology context, identifying multiple areas of new bone growth adjacent to the degradation layer of Mg-based implants. This newly formed bone, characterized by low mineralization, is distinctly highlighted in bright blue within the 3D virtual staining. One such region is visible across all three orthogonal planes, as indicated by the red arrows. Upon closer re-inspection of the original input 3D CT dataset, the prediction was supported by correspondingly lower greyscale values in the same region, although these features were not immediately apparent.

## 4. Conclusions and outlook

This work presents one of the first known demonstrations of virtual staining for 3D X-ray histology, enabled by a modified CycleGAN model adapted for paired SR-µCT and histology data of bone-implant systems. Through the integration of pixelwise supervision and greyscale loss terms, the model learns to synthesize artificially stained images from greyscale slices, bridging structural and chemical imaging modalities. Within the bone-implant context, the network captures both broad tissue organization and fine histological features, such as bone lacunae and degradation layers surrounded by new bone tissue, highlighting its potential for biomedical interpretation. Some colour mismatches, particularly in soft tissue regions, indicate limited generalizability to broader histology datasets. Addressing this may require advanced colour normalization, larger and more diverse training data, and progress on the wider challenge of colour standardization in digital pathology.

When compared against both standard CycleGAN and Pix2Pix baselines, the modified CycleGAN was shown to outperform in terms of structural similarity, perceptual fidelity and peak signal-to-noise ratio, as confirmed through both qualitative visual comparisons and quantitative metrics (SSIM, PSNR, LPIPS). In contrast to Pix2Pix, the supervised CycleGAN framework in particular demonstrated its suitability for cross-modality image pairs characterized by imperfect alignment at the pixel level, as is frequently the case in biomedical imaging applications. The standard CycleGAN without supervisory loss terms performed poorly, indicating that paired data are necessary for effective training in this specific application and domain. However, even with partial misalignment, the acquisition of paired data remains a bottleneck. Further studies could explore hybrid model architectures that incorporate unpaired data, such as the approach by Tripathy *et al.* (2019 ▸), which initially trains on paired datasets before refining with unpaired samples.

At the whole-slide image level, the method enables volumetric inference for µCT-based virtual histology at resolutions greater than 5 µm. Although this remains below the quality of conventional digital pathology slides, it is suitable for the volume sizes typically used in µCT studies. We successfully tested the trained forward model on a µCT stack, demonstrating its ability to generate virtual staining consistently across a full 3D dataset. Future work will focus on evaluating model performance at higher resolutions, closer to original histology quality, which will likely require interpolating CT data to upscale the training input. This could effectively transform the model into a super-resolution generator and enable deeper investigation into its spatial frequency response. Data augmentation strategies that incorporate artificial noise and smoothing filters could additionally improve robustness.

A further extension could include the integration of segmentation labels such as those used in 3D µCT analysis (Baltruschat *et al.*, 2021 ▸), supporting both evaluation and conditional training in future 3D applications. Quantitative evaluation could additionally include comparison with classical methods, for example the joint-histogram-based multi-class bone tissue segmentation reported by Rodgers *et al.* (2022 ▸).

Finally, to enhance clinical relevance, collaboration with medical experts will be crucial to ensure biological staining specificity and accuracy in digital pathology applications. We also plan to extend testing to other tissue types and applications, including datasets acquired with laboratory-based µCT systems, thereby broadening the model’s utility and generalizability. Expanding the dataset with more samples and additional staining types would further support these goals.

## 5. Related literature

The following references, not cited in the main body of the paper, have been cited in the supporting information: Nieuwenhuizen*et al.* (2013 ▸); Rieger *et al.* (2024 ▸).

## Supplementary Material

Sections S1 to S3 with Figs. S1 to S5. DOI: 10.1107/S1600577526004340/mo5315sup1.pdf

## Figures and Tables

**Table 1:** Evaluation of models with median values of the metrics SSIM, LPIPS and PSNR for training (from 40 WSI samples), validation (10 WSI samples) and testing (3 WSI samples). The top values are highlighted with bold text

	Metrics (training)			Metrics (validation)			Metrics (testing)		
Model	SSIM ↑	LPIPS ↓	PSNR ↑	SSIM ↑	LPIPS ↓	PSNR ↑	SSIM ↑	LPIPS ↓	PSNR ↑
Mod. CycleGAN (our model)	**0.63**	**0.12**	**17**	**0.60**	**0.16**	**15**	**0.59**	**0.16**	**15**
Pix2Pix	0.24	0.15	16	0.21	0.17	14	0.22	0.22	**15**
Std. CycleGAN + inv. CT input	0.27	0.25	13	0.31	0.25	13	0.33	0.25	12
Std. CycleGAN	0.15	0.44	5	0.18	0.43	5	0.20	0.43	5

## Data Availability

The data that support the findings of this study are available upon reasonable request from the authors.
