# Supplementary material for: Paired CycleGAN-based virtual staining for 3D X-ray histology of bone-implant systems
Source: J Synchrotron Radiat. 2026 Jun 5;33(Pt 4):1110–25. doi: 10.1107/S1600577526004340 (PMC13344548; doi:10.1107/S1600577526004340)
Supplement: Supplementary file 1 [file s-33-01110-sup1.pdf]

JOURNAL OF SYNCHROTRON RADIATION

Volume 33 (2026)

Supporting information for article:

Paired CycleGAN-based virtual staining for 3D X-ray histology of bone-implant systems

Sarah C. Irvine, Christian Lucas, Diana Krüger, Bianca C. Guedert, Julian Moosmann and Berit Zeller-Plumhoff

Supplementary Information

Supplementary document accompanying the manuscript: 'Virtual staining for 3D X-ray histology of bone implants'.

S1. TOLUIDINE BLUE HISTOLOGY DATA RESULTS

A range of examples from each of the training, validation and testing inference results from the modified paired CycleGAN model are shown in Figures S1a, S1b and S1c. The alloy material of each screw implant is given within the caption. The output WSIs generated through overlapping patch-based inference are displayed without sample masking. Consequently, we can observe various background effects such as tiling artefacts, and predictions based on the walls of the sample holder (seen as straight or circular depending on the cylinder-to-slice geometry). Sample 1 of each subset were previously shown in Figure 5 of the manuscript.

S2. H&E HISTOLOGY DATA RESULTS

In this section we describe the results of application of our paired models to a smaller, secondary dataset stained with H&E.

S2.1. H&E Datasets

For the secondary dataset stained with H&E, a total of 11 co-registered µCT and histology partial WSI pairs were able to be collated for this project. These comprised 3 samples containing Mg (-based implants), 6 containing Ti and 2 containing PEEK. We used the same hyperparameters for the modified CycleGAN and Pix2Pix as determined through validation performed on the Toluidine Blue dataset, but the models were re-initialised from scratch without pre-training before introducing the H&E dataset. For this qualitative demonstration, training was performed on 8 WSI pairs (2 Mg, 5 Ti and 1 PEEK) with the remaining 3 pairs for validation/testing (1 each of Mg, Ti and PEEK).

Due to the laser cutting process for this dataset (as opposed to the physical cutting and grinding of the Toluidine Blue (see Section 2.1 of the manuscript) the residual screw alloy components are missing from the histology slices. Consequently the screw appears white instead of black (with the one exception of Sample 2 in Figure S3b, which retains a half section of residual Mg-based alloy in black). There are a few missing sections of tissue too, resulting also in corresponding white regions. The fields of view (FOV) of these partial WSIs are limited and non-rectangular; regions outside of the existing stitched data are depicted as black. Sample correspondence maps were created to reflect the stitched FOVs.

S2.2. Description of H&E results

Three examples from each of the training, and validation/testing inference results from the modified CycleGAN model are shown in Figures S2 (masked view) and S3 (unmasked view). A direct comparison is more easily made with the masked view. In the unmasked view, the incompleteness of the tiled field of view causes uncertainty outside of these stitched regions (since the sample correspondence mask is also applied to the loss terms during training). This yields false areas of white in the generated µCT images and black in the histology. On the other hand, the generated histology images are reasonably able to be predicted across the full rectangular FOV provided by the input µCT slice.

In Figure S2, we see a promising level of agreement between real and generated histology images in terms of structure and colour. Although the dataset is small, these results demonstrate that our modified CycleGAN model may be reasonably applied to larger H&E datasets as well as Toluidine Blue, suggesting its utility as a general stain-appropriate model. However, it is not really possible to judge the interpretability of the trained model results in terms of previously mentioned features such as degradation layer and new bone formation for a number of reasons. Firstly, the histology quality is not as good, particularly surrounding the region which contained the screw implant in several samples (see for example the missing tissue sections in Sample 2 of Figure S2b.) Secondly, the degradable samples were here under-represented, with only 3 total Mg samples present in which to observe the degradation layers. In those samples, the H&E stain differentiates less strongly between the degradation layer, new bone and even some parts of soft

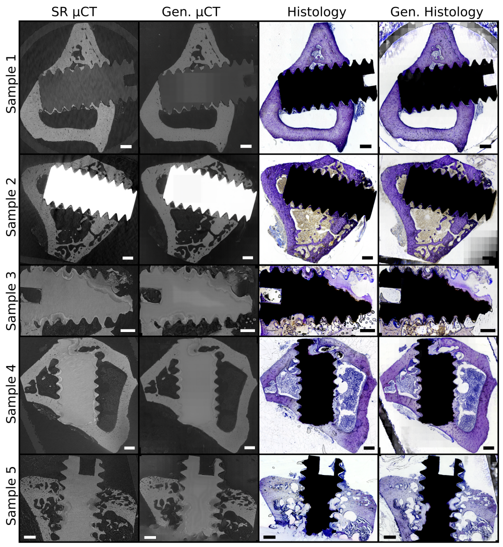

(a) Training results (5 of 40 WSI samples)

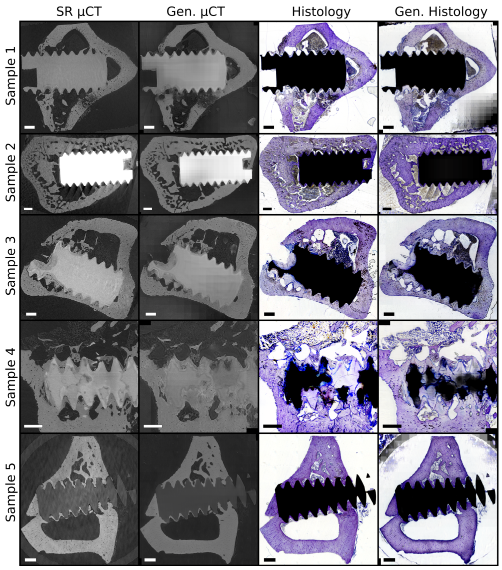

(b) Validation results (5 of 10 WSI samples)

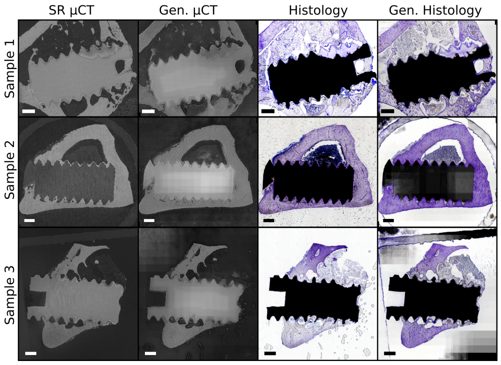

(c) Test results (3 of 3 WSI samples)

Fig. S1. The modified CycleGAN model results (with WSI output). In training: Sample 1 = PEEK, Sample 2 = Ti, Sample 3 = Mg, Sample 4 = Mg, Sample 5 = Mg. In validation: Sample 1 = Mg, Sample 2 = Ti, Sample 3 = Mg, Sample 4 = Mg, Sample 5 = PEEK. In testing: Sample 1 = Mg, Sample 2 = PEEK, Sample 3 = Mg. Scalebars represent 500 µm.

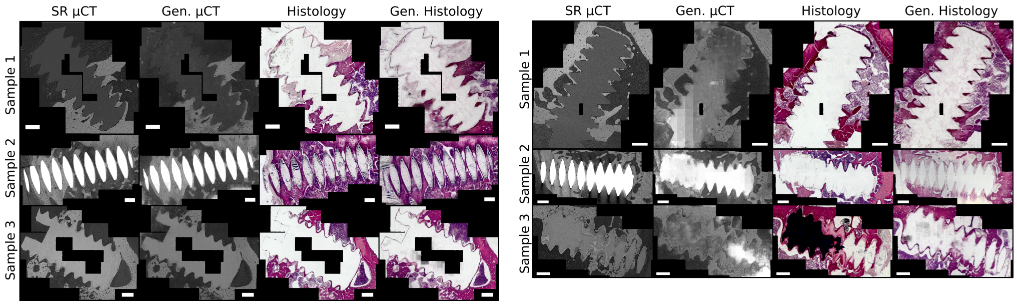

(a) training results (3 of 8 WSI samples) **(b)** validation/testing results (3 of 3 WSI samples)

Fig. S2. Masked views of the inference results of the modified CycleGAN model applied to the secondary H&E stained dataset (masked view). In training: Sample 1 = PEEK, Sample 2 = Ti, Sample 3 = Mg. In validation/testing: Sample 1 = PEEK, Sample 2 = Ti, Sample 3 = Mg. Scalebars represent 500 µm.

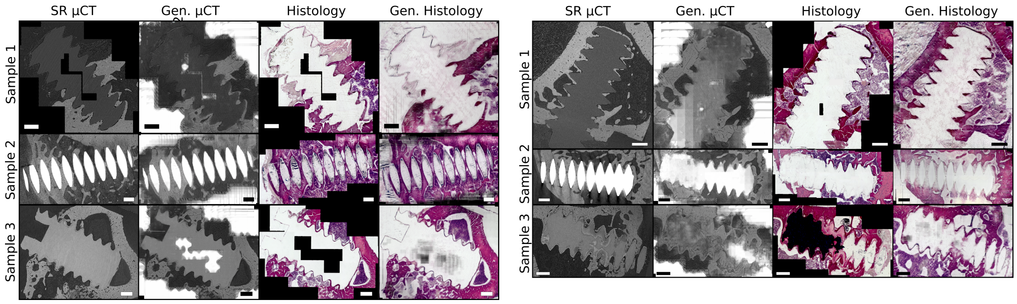

(a) training results (3 of 8 WSI samples) **(b)** validation/testing results (3 of 3 WSI samples)

Fig. S3. Unmasked views of the inference results of the modified CycleGAN model applied to the secondary H&E stained dataset. In training: Sample 1 = PEEK, Sample 2 = Ti, Sample 3 = Mg. In validation/testing: Sample 1 = PEEK, Sample 2 = Ti, Sample 3 = Mg. Scalebars represent 500 µm.

tissue, all of which are observed to present in various shades of pale pink, as compared to the deep pink-red of the dense bone material.

S2.3. Qualitative comparison of our model vs Pix2Pix

In Figure S4 we qualitatively compare the H&E stain-based inference results of our modified paired CycleGAN model with those of Pix2Pix, with an example validation/testing WSI plus 256 × 256 pixel ROI. The generated histology images are shown as masked with the sample correspondence map.

It is clear that our model-generated histology image is a more accurate prediction of the real histology image. In both models, the soft tissue (coloured purple) is not very sharply reproduced, but they are better defined by the CycleGAN model. Pix2Pix also fails to give a strong delineation between the region belonging to the implant (here PEEK-based, which has osseointegrated) and the surrounding bone. Interestingly, the Pix2Pix generated regions of dense bone feature a high degree of style-based realism in the way it has reproduced the white cracks characteristic of the real histology dense bone. However, these cracks were formed in the bone sections after the whole volume tomography acquisition process and do not correspond to the real µCT data. These may be classed as hallucinations by the GAN, produced in an effort to satisfy the discriminator.

S3. RELATIVE RESOLUTION OF THE GENERATED HISTOLOGY IMAGES

We performed preliminary image spatial resolution measurements on the input/output Toluidine Blue-stained data, in a study of our modified CycleGAN model. Measurements were based on the Fourier ring correlation method [1, 2], and sampling was performed with 256 × 256 pixel patches across the WSI images, excluding the masked areas. Absolute values in units of pixels were

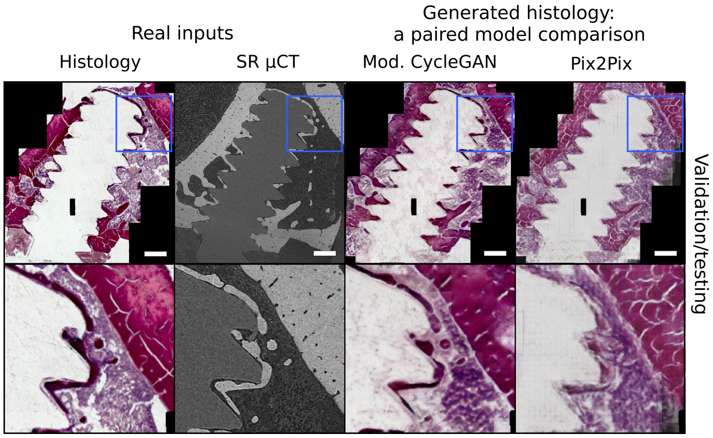

Fig. S4. Comparison of inference results from the modified CycleGAN and Pix2Pix paired model with the H&E stained data validation/testing set. Including masked WSI and 256 × 256 pixel ROI examples. The scalebar in each WSI represents 500 µm.

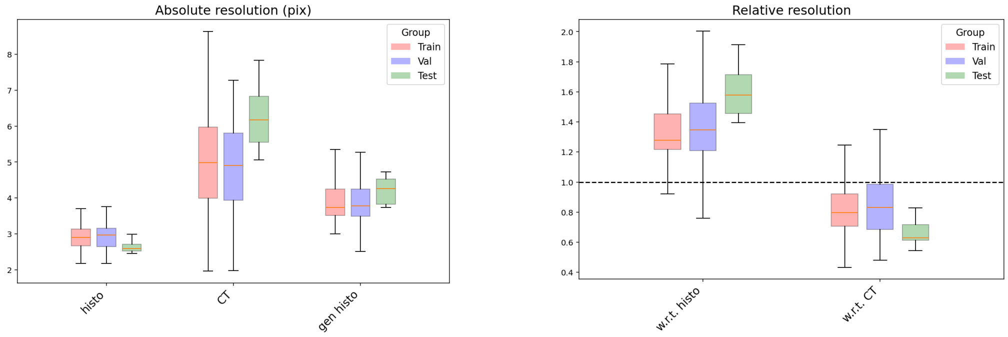

(a) **(b)**

Fig. S5. Box plots of the measured resolution of the generated histology compared to the input real histology and CT data, shown in both absolute values (in units of pixels) in (a), and in relative terms in (b).

calculated for both the generated histology images as well as the original input CT and histology images, from which we also derived a spatial resolution value relative to both inputs. The relative fraction was calculated per image pair due to high levels of image inhomogeneity. The results of both are shown with two plots in Figure S5, for each of the train, validation and test groups. In absolute resolution terms, the input CT was generally considerably lower in resolution than the corresponding input histology (with a mean of around 5-6 pixels, compared to less than 3 pixels for the histology), whilst the generated histology resolution was somewhere in-between (around 4 pixels). In relative terms, for the training and validation sets the generated histology resolution was found to be on average 1.35× the resolution of the histology and 0.8 × the resolution of the CT. In the three test WSI pairs, the input CT images were notably worse in resolution than the 50 train+validation pairs, and this effect became more pronounced (with a value of less than 0.7 × the resolution of the CT). Note however that it is difficult to accurately measure the resolution due to various high-frequency artefacts present in the images which may influence the result. It is also not straightforward to directly compare the signal-to-noise distributions of the histology and CT images since they are so different. A more comprehensive study may be performed at a later stage, perhaps in combination with a higher-resolution dataset (involving less-downsampling of the original WSI histology images).

REFERENCES

1. R. P. Nieuwenhuizen, K. A. Lidke, M. Bates, *et al.*, "Measuring image resolution in optical nanoscopy," Nat. methods **10**, 557–562 (2013). Publisher: Nature Publishing Group US New York.
2. B. Rieger, I. Droste, F. Gerritsma, *et al.*, "Single image Fourier ring correlation," Opt. Express **32**, 21767 (2024).
